# Effectiveness of a parent‐targeted video on neonatal pain management: Nonrandomized pragmatic trial

**DOI:** 10.1002/pne2.12023

**Published:** 2020-05-06

**Authors:** Ligyana Korki de Candido, Denise Harrison, Maria de La Ó Ramallo Veríssimo, Mariana Bueno

**Affiliations:** ^1^ School of Nursing University of Ottawa Ottawa Ontario Canada; ^2^ University of Ottawa and Children's Hospital of Eastern Ontario Ottawa Ontario Canada; ^3^ Department of Maternal‐Child and Psychiatric Nursing School of Nursing of the University of São Paulo São Paulo Brazil; ^4^ The Hospital for Sick Children Child Health Evaluative Sciences Toronto Ontario Canada

**Keywords:** educational technology, infant, neonatal nursing, newborn, pain, parents

## Abstract

The “Be sweet to babies” video is a knowledge translation tool targeted at parents on the use of analgesic strategies during painful procedures performed in neonates. This study aimed to evaluate the effectiveness of the Portuguese version of this video on maternal involvement in neonatal pain management during the newborn screening blood test. Nonrandomized, pragmatic clinical trial. The study was conducted in a rooming‐in unit. All mothers received a pamphlet regarding neonatal pain management and were invited to participate in daily education sessions conducted by nurses, which included the video. The intervention group included mothers who voluntarily watched the video, while the control group was comprised of mothers who did not attend the education sessions or left the session before watching the video. Data were collected by interviews conducted by the research team. Descriptive and inferential analysis considered a confidence interval of 95%. A total of 73 mothers were included in the study. Analgesic strategies were used in 14 (40%) of the procedures in the intervention group and 9 (24%) in the control group, a clinically important difference of 16% points between groups, although no statistically significant difference was found (*P* = .13). Breastfeeding was the most commonly used strategy. Watching the video increased the chance of implementing analgesic strategies by 2.1 times (*P* = .19), while nurses suggesting the use of analgesia increased this chance by 5.5 times (*P* = .006). Although no statistical significance was found, the results suggest the clinical significance and feasibility of the “Be sweet to babies” video as a KT tool targeted at parents on neonatal pain management during nonurgent painful procedures. In addition, maternal involvement in pain care significantly increased when pain relief strategies were recommended by nurses, which suggests that nurses have a key role in facilitating parental participation.

## 1 INTRODUCTION

Nearly all newborns experience painful procedures as part of routine newborn screening (NBS)^1^ and vaccination^2^ and sick hospitalized infants experience many more painful procedures as part of their medical care.^3^, ^4^ Poorly treated pain in early life can affect newborns’ brain growth and development, as well as the way they experience pain later in life.^5^, ^6^, ^7^, ^8^ However, published reports show inconsistent use of analgesia in diverse clinical care settings.^3^, ^4^, ^9^ Involving parents in neonatal pain management practices may improve the use of analgesic strategies during nonurgent painful procedures.^10^, ^11^, ^12^, ^13^

The “Be sweet to babies” video is an evidence‐based knowledge translation (KT) tool which was co‐produced with parents and targeted at parents to assist nurses in providing information according to parents’ preferences and needs. It demonstrates the use and effectiveness of breastfeeding^14^ skin‐to‐skin care (SSC)^15^ and small volumes of sucrose^16^, ^17^ or glucose^16^, ^18^ as analgesic strategies during blood sampling, uses lay person's language, and encourages parents to ask healthcare professionals (HCP) about ways they can help their babies during painful procedures.^19^ The Portuguese language version of the video is 6‐minutes long. It has been available online since 2014, and was updated in 2016 to reflect recommendations and endorsement from Baby‐Friendly Hospital Initiative (BFHI)^20^ concerning use of sucrose or glucose and pacifier (https://youtu.be/ZGLSNdYtppo ).

The video has been evaluated by parents and HCP as useful, easy to understand, applicable in real scenarios, persuasive, and an ideal length.^19^, ^21^, ^22^, ^23^, ^24^ However, the impact of this video on the use of analgesic strategies during painful procedures performed in neonatal care units is still unknown.^22^ ^,^ ^23^

We hypothesized that mothers exposed to the Portuguese version of the video would be more participative in using analgesic strategies during nonurgent painful procedures performed in neonates. Therefore, this study aimed to evaluate the effectiveness of the Portuguese version of the "Be sweet to babies" video on maternal involvement in neonatal pain management during the NBS blood test.

## 2 METHODS

### 2.1 Design

This is a nonrandomized, nonblinded, pragmatic clinical trial conducted in the rooming‐in postnatal maternity ward of an affiliated hospital of the University of São Paulo, Brazil. This institution is a public teaching hospital and certified by the BFHI^20^ since 2007. After birth, mothers and newborns are admitted to the rooming‐in unit, except those with clinical complications that require more complex care in other units.

At the time of this study, there were no educational activities targeted at parents concerning pain management during NBS and there was no guideline on procedural pain management in the unit. However, all nurses had already watched the “Be sweet to babies” video in a previous study.^21^ The study protocol was approved by the ethics review boards of the affiliated university and hospital (protocols: 2.026.678 and 2.038.006, respectively) and registered in the Brazilian Registry of Clinical Trials platform (protocol: RBR‐9595ds). Informed consent was obtained from the eligible mothers by research team members prior to their inclusion in the study during the first 24 hours of hospitalization in the unit, except for mothers who were physically or emotionally unwell. The informed consent form used lay language to inform mothers about the aim and design of the study, including information about the evaluation of the educational intervention and voluntary inclusion in the control (CG) or intervention groups (IG).

### 2.2 Participants

Mothers admitted to the rooming‐in unit during the study period, over the age of 16 years old, able to give informed consent, able to understand, read and write in Brazilian Portuguese, and whose newborns, born at any gestational age, were able to be breastfed, positioned SSC, or receive the sweet‐tasting solution of 25% glucose were eligible. After enrollment, the following exclusion criteria were applied: failure to successfully collect blood for NBS on the first heel lance attempt; NBS collected through venipuncture instead of the standard procedures of heel lance for capillary blood sampling; NBS collected without mothers’ presence; and newborns transferred to another unit.

### 2.3 Enrollment, intervention, and data collection procedures

Data collection occurred during the month of July 2017, during 17 uninterrupted days. Upon admission, all women received a pamphlet with the same information as the “Be sweet to babies” video to ensure the opportunity to receive information about effective pain‐reduction strategies. All women were invited to participate in daily postnatal education sessions conducted by nurses, which addressed postpartum and newborn care, and included the “Be sweet to babies” video as the last topic addressed. Nurses did not provide further opportunities to explain or discuss the content of the video.

The IG was comprised of mothers who voluntarily watched the video (intervention), while the CG was comprised of mothers who did not attend the education sessions or left the session before watching the video. All mothers in the CG were invited to watch the video after completing the research questionnaire. Participants were not blinded due to the impossibility of concealing the proposed intervention in the routine of the rooming‐unit.

After 48 hours of birth, newborns underwent NBS. Lastly, mothers were invited to answer a questionnaire administered by the research team. The questionnaire was developed based on prior studies.^25^ ^,^ ^26^ It was comprised of 20 questions, including demographic characteristics of mothers and newborns, mothers’ participation in the educational sessions, and mothers’ perception on their involvement on neonatal pain management during the NBS blood test. The prevalence of the use of analgesic strategies during the NBS blood test was the primary outcome. Secondary outcomes included the following: type of analgesic strategies used (breastfeeding, SSC, sweet‐tasting solution, and other), nurses’ suggestion of analgesic strategies use (yes/no question); and other types of mothers’ or other family members’ involvement during the NBS blood test (observing, holding, and advocating for pain management). Infant characteristics such as gestational age at birth and number of previous painful procedures were reviewed in the newborns’ medical chart. Painful procedures were defined as skin‐breaking procedures such as injection, venipuncture, and heel lance performed due to vaccination (hepatitis B and BCG‐tuberculosis) and blood collection for bilirubin test, capillary blood glucose monitoring, and other tests. Although the use of analgesic strategies during these previous painful procedures was not investigated in this study, some newborns were breastfed during the procedures, according to mothers’ and nurses’ reports.

### 2.4 Sample size

Sample size was based on a previous pilot study^27^ ^,^ with the aim of 80 participants, a level of significance (α) of 5%,power of (1 − β) 90%; and absolute difference of 39% in the use of analgesic strategies between groups and a loss to follow‐up rate of 20%.

### 2.5 Data analysis

Data were stored in a Microsoft Excel for Windows® (version 2011) spreadsheet, and descriptive and inferential analysis were performed in SPSS version 17.0, considering a confidence interval (CI) of 95%. Pearson's chi‐square (two‐sided) and Fisher's exact test (two‐sided) were used to analyze categorical variables, and the Paired t test (two‐sided) was used to compare the means of the quantitative variables between groups. In addition, a logistic regression evaluated the association of the primary outcome with the following variables: visualization of the video, pamphlet reading, number of children, number of previous painful procedures, father's and other relative's presence during NBS, and nurses’ suggestion of analgesic strategies’ use.

## 3 RESULTS

**FIGURE 1:**
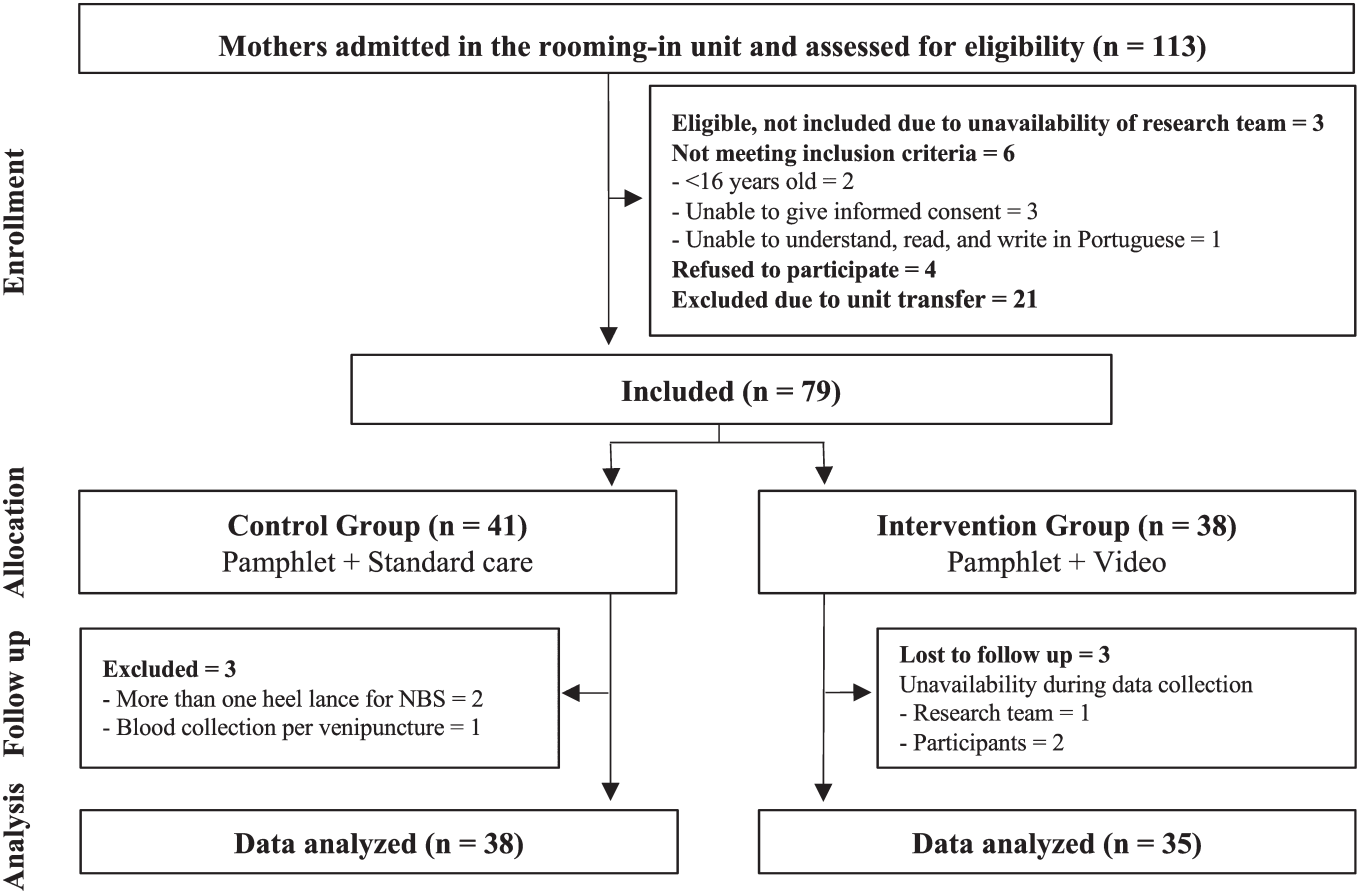
Flow diagram of the inclusion of participants in the study

During the study period, 113 mothers with their newborns were admitted in the unit. From these, 79 were included in the study. After exclusions and losses to follow‐up, data analysis comprised 73 participants Figure 1 **.**

Demographic characteristics of participating mothers were similar between groups except for the mean number of children, which was higher in the CG Table 1.

**TABLE 1:** Demographic characteristics of the 73 mothers included in the study

Variables	Control Group (n = 38)		Intervention Group (n = 35)		*P*
	n	%	n	%	
Marital status					
Married/Common law	31	82	31	89	.40*
Single	7	18	4	11	
Type of delivery					
Vaginal	22	58	21	60	.53**
Cesarean	12	32	13	37	
Forceps	4	10	1	3	

	Mean (SD)	Median	Mean (SD)	Median	*P*
Age (years)	26.9 (5.3)	27.0	26.5 (6.4)	25.0	.75***
Education (years)	11.0 (3.1)	11.0	10.5 (2.5)	11.0	.49***
Number of children	2.2 (1.0)	2.0	1.7 (0.7)	2.0	.013***

* Pearson's chi‐square;

** Fisher's exact test;

*** t test.

Newborns’ demographic and clinical characteristics were similar Table 2. Prior to NBS blood test, all newborns underwent at least two painful procedures due to vaccination (hepatitis B and BCG‐tuberculosis). Less than half of newborns underwent venipuncture and heel lance due to blood collection for bilirubin test and capillary glucose monitoring. The number of previous painful procedures ranged from two to 16 per newborn.

**TABLE 2:** Newborns’ demographic and clinical characteristics

Variables	Control Group (n = 38)		Intervention Group (n = 35)		*P*
	n	%	n	%	
Sex					.90*
Female	19	50	18	51	
Male	19	50	17	49	
Breastfeeding					1.00**
Exclusive	36	95	34	97	
Supplementary feeding	2	5	1	3	
Previous painful procedures					
Vaccines	38	100	35	100	‐‐
Blood collection	17	45	13	37	.51*

	Mean (SD)	Median	Mean (SD)	Median	*P*
Gestational age at birth (weeks)	39.9 (1.3)	40.0	40.0 (0.9)	40.0	.65***
Number of previous painful procedures	3.4 (2.6)	2.0	3.2 (1.9)	2.0	.72***

* Pearson chi‐square;

** Fisher's exact test;

*** t test.

The pamphlet on neonatal pain management was read by 16 (42%) mothers in the CG and 18 (51%) in the IG (*P* = .30). Fathers or other relatives accompanied mothers during 14 (37%) NBS blood tests performed in the CG and 15 (43%) in the IG (*P* = .87).

### 3.1 Pain management during NBS blood test

There was no statistically significant difference between groups in terms of mothers’ involvement in neonatal pain management. Analgesic strategies were used in 9 (24%) of the procedures in the CG and 14 (40%) in the IG (*P* = .13), an absolute difference of 16% between groups. Breastfeeding was the most commonly used strategy in both groups. SSC and non‐nutritive sucking (NNS) were provided only in the IG. Watching the video doubled the chance of using any analgesic strategy, breastfeeding 1.5 times, SSC 4.6 times, and NNS 2.2 times Table 3.

**TABLE 3:** Prevalence of use of analgesic strategies during newborn screening blood test

Variables	Control Group (n = 38)		Intervention Group (n = 35)		*P*	OR	CI 95%^a^
	n	%	n	%			
Analgesic strategies	9	24	14	40	.13*	2.1	0.78‐5.89
Breastfeeding	9	24	11	31	.09*	1.5	0.53‐4.15
Skin‐to‐skin care	0	0	2	6	.24**	4.6	0.20‐105.77
Sweet‐tasting solution	0	0	0	0	‐	‐‐	‐‐
Non‐nutritive sucking	0	0	1	3	.49**	2.2	0.07‐68.75

^a^ CI related to Odds ratio (OR).

* Pearson chi‐square.

** Fisher's exact test.

For infants who did not receive any pain management strategies, most of their mothers (CG – 29/76%; IG – 20/57%; *P* = .13) reported they held their newborns during NBS. Only one participant in the IG (3%) and none in the CG reported observing the procedure while the newborn was lying down on a crib (*P* = .48). There was no significant difference between groups regarding nurses’ encouraging parents’ participation (*P* = .57). Nurses suggested breastfeeding during NBS in approximately half of the cases (CG – 17/45%; IG – 18/51%), although this suggestion was not heeded by all mothers. Three (9%) mothers from the IG and none in the CG reported advocating for the use of analgesic strategies (*P* = .10).

In an adjusted analysis, nurses suggesting the use of analgesia were the only significant association (*P* = .006), increasing 5.5 times the chance of implementing analgesic strategies during NBS. Watching the video increased the chance of implementing analgesic strategies by 2.1 times Table 4.

**TABLE 4:** Logistic regression model of use of analgesic strategies during newborn screening blood test (n = 73)

Independent variables	B	*P*	OR	CI 95%
Video	0.76	.19	2.1	0.69‐6.61
Pamphlet	−0.47	.43	0.6	0.19‐2.00
Number of children	−0.38	.25	0.7	0.36‐1.31
Number of previous painful procedures	−0.07	.68	0.9	0.67‐1.30
Father's presence	−1.09	.11	0.3	0.09‐1.26
Other relative's presence	0.57	.58	1.8	0.23‐13.60
Nurse's suggestion of analgesic strategies use	1.70	.006	5.5	1.64‐18.43
Constant	−0.66	.51	0.6	‐

After participating in this study, all mothers in the IG and the majority of mothers in the CG (36/95%) intend to use breastfeeding, SSC, or sweet‐tasting solutions in future painful procedures (*P* = .49), such as vaccinations and blood collection. Three (8%) mothers in the CG refused to watch the video after answering the questionnaire. No adverse events associated with the video or the use of analgesic strategies were reported or observed.

## 4 DISCUSSION

This study aimed to evaluate the effectiveness of the Portuguese version of the "Be sweet to babies" video on neonatal pain management by assessing the prevalence of use of evidence‐based recommended analgesic strategies during NBS blood test.

Although no statistically significant difference was found between groups regarding the use of analgesic strategies during the NBS (*P* = .134), the absolute difference of 16% could be considered as clinically important. Based on the study conducted by Shah, Ipp, Sam, Einarson & Taddio,^28^ a minimal difference of 13% was accepted as clinically important for neonatal pain reduction, according to parent's perceptions. There has been recent recommendation on interpretation of *P* values since they do not provide information on the magnitude of an effect, with special attention to the health relevance of the findings.^29^, ^30^ Decision makers also need to consider the feasibility and costs to implement educational materials in clinical practice.^31^ In our study, the “Be sweet to babies” video was shown to be a feasible intervention embedded into the unit's routine with a relevant impact in clinical practice. Moreover, the video is freely available online in multiple languages, which reduces potential associated costs with its use.

To date, few studies have evaluated the effectiveness of instructional videos on the use of analgesic strategies during painful procedures. A Canadian randomized controlled trial (RCT)^26^ which included 174 expectant mothers, evaluated a complex intervention, comprised of a video, a presentation, a pamphlet, and a question and answer period, on infant's pain management during vaccination. Similar to our study, there was an absolute increase of 17% in the use of analgesic strategies (GC – 17% and IG – 34%,*P* = .01) and breastfeeding during vaccination was the most commonly used strategy. Conversely, sucrose was the second most commonly used strategy, while sweet‐tasting solutions were not used at all in our study, despite the availability of 25% glucose in the unit for painful procedures.

In two additional RCTs conducted in Canada, the effectiveness of videos in complex and active interventions (eg, including a poster and a pamphlet, offering instructions, and answering questions) increased the use of any pain relief strategies during infant's vaccination by 10%^32^ and 54%.^33^ Both studies reported more frequent use of breastfeeding and holding, respectively. Although recommended for older infants, especially during immunization, upright holding is considered to provide comfort rather than analgesia for neonates and is reported as low‐quality evidence in a systematic review of nonpharmacological strategies for management of procedural pain.^34^ Therefore, holding was not considered as an analgesic strategy in our study.

The “Be sweet to babies” video recommends the use of breastfeeding, SSC, and sweet‐tasting solutions, which are effective, low cost, safe, and easy to apply interventions. Breastfeeding was expected to be the most commonly used analgesic strategy in both groups in our study, since it was conducted in a rooming‐in unit certified by the BFHI that aims to promote, protect, and support breastfeeding.^20^ Accordingly, although glucose 25% was available in the unit, nurses could be less likely to provide or support its use, compared to breastfeeding and SSC, because of an erroneous belief that sweet‐tasting solutions are not allowed by the BFHI, as reported in a previous study conducted in this same setting, which assessed nurses’ perceptions on the “Be sweet to babies” video.^21^ In fact, the video has been endorsed by the BFHI on the use of small volumes of sweet‐tasting solutions, with or without NNS, for neonatal pain management when newborns are unable to be breastfed or positioned in SCC.^23^ It is worth noting that one mother in the IG reported the use of NNS alone as an analgesic strategy during the NBS blood test. However, evidence on the analgesic effects of NNS alone is reported as low‐quality evidence^34^ ^,^ being recommended in combination with sucrose^17^, ^35^ or glucose^18^ due to the synergic effect of these interventions.

In regard to the positive impact of nurses encouraging parents in the use of analgesic strategies during NBS, our findings confirm that nurses have a key role in facilitating parental involvement in neonatal pain management^10^ ^,^ ^11^, ^36^ and that educational interventions targeted at parents also need to involve nursing staff to optimize their success.^23^, ^32^ Despite the importance of the collaboration between nurses and parents, nurses however do not always provide information, encouragement, and opportunities for parents to participate on newborns’ pain care.^9^, ^36^, ^37^, ^38^ According to the participating mothers, nurses suggested the use of analgesia in only half of the NBS performed in this study. In addition to the need of educating nurses to improve neonatal pain management practices, the availability of effective KT tools targeted at parents in neonatal care units may assist nurses and help parents to advocate for use of effective pain‐reduction strategies during painful procedures, including capillary blood sampling for NBS.

Furthermore, parents increasingly search for health information on the internet^39^, ^40^, ^41^ which reinforces the need to provide high‐quality evidence‐based online resources on neonatal pain management^39^, ^41^ ^,^ as well as evaluate their impact in clinical practices. Parents and HCP have been accessing the “Be sweet to babies” video on social media platforms in many languages across many countries, with positive results in terms of knowledge acquisition, acceptability, feasibility, and intention to use the strategies for future painful procedures.^19^ ^,^ ^24^ In this study, the Portuguese version of the video was also determined to be potentially effective, however, pain management strategies were still only used in 40% of cases in the IG.

After participating in this study, the majority of mothers reported the intention to use breastfeeding, SSC, or sweet‐tasting solutions in future painful procedures performed in their infants, such as vaccination and blood collection. It is worth highlighting that the analgesic effects of breastfeeding^42^ and sweet‐tasting solutions^43^ persist up to one year old, which indicates that the video may impact pain management practices beyond hospital discharge. Further studies are recommended to ascertain effectiveness of the video in improving pain management during early childhood vaccinations and other painful procedures.

### 4.1 Strengths and limitations

To the authors’ knowledge, this is the first study that investigates the effectiveness of an educational video targeted at parents on the use of analgesic strategies in the Brazilian neonatal context. Its pragmatic design enabled the evaluation of this KT tool in a real scenario, and results are therefore likely to be generalizable to other rooming‐in units around the world. A pilot study was conducted in the same setting (n = 23) to assess the feasibility of the full‐scale trial, elucidating the need to investigate nurses’ suggestion of use of analgesic strategies during the NBS blood test. Furthermore, for ethical reasons, mothers from the CG received a pamphlet on neonatal pain management upon their admission in the unit and had an opportunity to watch the video after participating in the study.

High‐quality evidence supports the effectiveness and safety of breastfeeding, SSC, and sweet‐tasting solutions in reducing pain scores during nonurgent minor painful procedures.^14^, ^15^, ^16^, ^17^, ^18^ Therefore, the authors decided to investigate a process indicator (the use of analgesic strategies) as the primary outcome.^44^ In addition, neonatal pain assessments were conducted and documented in the clinical charts of all included babies by the nurses who performed the procedures, as part of the institution's current practices.

The nonblindness of participants and nonrandomized design are study limitations; however, this was a pragmatic trial, conducted in an ethical responsible manner. The mean number of children may have influenced mothers' allocation into groups since 16 mothers, who did not participate in the postnatal educational sessions, reported that they had already gained enough knowledge and experience caring for their other children. Regarding mothers who participated in the educational sessions, knowledge acquisition may have been hindered due to the fact that the video was shown at the end of the sessions, following a large amount of information. In addition, the small sample size based on an absolute difference of 39% in the primary outcome between groups, which is higher than the average used in KT studies, may have resulted in the study being underpowered, influencing the statistical significance of the results. However, there were no other studies that investigated the effectiveness of instructional videos targeted at parents on neonatal pain management to guide the sample size estimation. The decision to estimate the sample size based on a pilot study also considered ethical aspects, feasibility, and resources available to conduct this research.

## 5 CONCLUSIONS

Although no statistical significance was found, the results suggest the clinical significance and feasibility of the “Be sweet to babies” video as a KT tool targeted at parents on neonatal pain management during nonurgent painful procedures. The video was simply inserted in a rooming‐in unit routine, increasing the prevalence of use of analgesic strategies by mothers during the NBS blood test. However, in our study, nurses suggesting the use of pain relief strategies had greater impact on mothers’ participation on neonatal pain management.

## CONFLICT OF INTEREST

The authors have no conflict of interest to declare.

## AUTHORS’ CONTRIBUTIONS

LC, MB, and DH have made substantial contributions to conception and design of the study. LC, MV, and MB have been involved in data analysis. LC has been involved in drafting the manuscript. All authors have been involved in critically reviewing and revising the manuscript for important intellectual content and have read and approved the final manuscript.
